# SNAI1 recruits HDAC1 to suppress *SNAI2* transcription during epithelial to mesenchymal transition

**DOI:** 10.1038/s41598-019-44826-8

**Published:** 2019-06-05

**Authors:** Vignesh Sundararajan, Ming Tan, Tuan Zea Tan, Jieru Ye, Jean Paul Thiery, Ruby Yun-Ju Huang

**Affiliations:** 1 Cancer Science Institute of Singapore, National University of Singapore, Center for Translational Medicine, 14 Medical Drive, MD6 #12-01, 117599 Singapore, Singapore; 2 Department of Biochemistry, Yong Loo Lin School of Medicine, National University of Singapore, 8 Medical Drive, MD7, #02-03, Singapore, 117597 Singapore; 3 Guangzhou Institute of Biomedicine and Health, Chinese Academy of Science, Guangzhou, People’s Republic of China; 4 CNRS Emeritus CNRS UMR 7057 Matter and Complex Systems, University Paris Denis Diderot, Paris, France; 5 INSERM UMR 1186, Integrative Tumor Immunology and Genetic Oncology, Gustave Roussy, EPHE, PSL, Fac. de Médecine - Univ. Paris-Sud, Université Paris-Saclay, 94805 Villejuif, France; 6 Department of Obstetrics and Gynaecology, National University Hospital of Singapore, 1E Kent Ridge Road, 119228 Singapore, Singapore; 7 Department of Anatomy, Yong Loo Lin School of Medicine, National University of Singapore, 4 Medical Drive, MD10 #04-01, Singapore, 117597 Singapore; 8 Present Address: School of Medicine, College of Medicine, National Taiwan University, No. 1 Ren Ai Road Section 1, 10051 Taipei, Taiwan Republic of China

**Keywords:** Gene silencing, Ovarian cancer

## Abstract

Aberrant activation of epithelial to mesenchymal transition (EMT) associated factors were highly correlated with increased mortality in cancer patients. SNAIL family of transcriptional repressors comprised of three members, each of which were essentially associated with gastrulation and neural crest formation. Among which, SNAI1 and SNAI2 were efficiently induced during EMT and their expressions were correlated with poor clinical outcome in patients with breast, colon and ovarian carcinoma. In an ovarian cancer cell lines panel, we identified that SNAI1 and SNAI2 expressions were mutually exclusive, where SNAI1 predominantly represses SNAI2 expression. Detailed analysis of *SNAI2* promoter region revealed that SNAI1 binds to two E-box sequences that mediated transcriptional repression. Through epigenetic inhibitor treatments, we identified that inhibition of histone deacetylase (HDAC) activity in SNAI1 overexpressing cells partially rescued *SNAI2* expression. Importantly, we demonstrated a significant deacetylation of histone H3 and significant enrichments of HDAC1 and HDAC2 corepressors in both E-box regions of *SNAI2* promoter. Our results suggested that SNAI1 repression on SNAI2 expression was predominantly mediated through the recruitment of the histone deacetylation machinery. Utilization of HDAC inhibitors would require additional profiling of SNAI1 activity and combined targeting of SNAI1 and HDACs might render efficient cancer treatment.

## Introduction

Epithelial-Mesenchymal Transition (EMT) is a reversible process, where epithelial cells lose apico-basal polarity and intercellular junctions to form invasive and motile mesenchymal cells or cells with the mesenchymal phenotype, express mesenchymal markers and acquire the front-rear polarity^1^. EMT process is conserved throughout evolution as it contributes to embryogenesis and organ development. In addition, EMT has been implicated during carcinoma progression such as mediating therapeutic resistance^2,3^. Several transcription factors essential during gastrulation have been shown to play central roles in orchestrating the EMT process.

The SNAIL family is the best studied EMT transcription factor^4^. *SNAI1* was first discovered as snail in *Drosophila* in 1984. Grau Y *et al*., found that mutants at the snail locus are zygotically embryonic lethal, though affecting dorsoventral patterning indicating that snail plays essential roles during embryogenesis^5^. *SNAI2* was first described by Nieto *et al*., ten years later than the discovery of *SNAI1*. In this study, *SNAI2* was named as slug and found to be critical in chick embryo mesoderm formation and neural crest emigration during gastrulation, evidenced by the inhibition of slug specifically impeded the normal change in cell behaviour^6^. Belonging to the zinc finger transcription factor family, SNAI1 and SNAI2 proteins are small in size while maintaining the ability of binding or recruiting several co-regulators. SNAI1 and SNAI2 share a conserved organization, given that both of them are composed of a group of 4 (SNAI1) to 5 (SNAI2) C2H2 type zinc fingers at C-terminal and a SNAG domain at N-terminal^7^. SNAI2 is characterized by a centrally located slug domain which is absent in SNAI1. The SNAG domain of SNAI1, has been shown to bind corepressor, such as HDAC1/2 for histone deacetylation^8^, the protein arginine methyltransferase 5 (PRMT5) for its nuclear translocation^9^, the coREST for the formation of SNAI1-LSD1-CoREST repressive complex^10^, and polycomb repressive complex 2 (PRC2) for gene repression^11^. In the case of SNAI2, co-repressors NCoR and CtBP1 interact with SNAG and SLUG domain of SNAI2, respectively^12^.

Being the prototype of the family, the regulation of *SNAI1* transcription has been extensively studied and reviewed^13,14^. The regulation of *SNAI2* transcription on the contrary is less characterized. The expression of *SNAI2* is known to be regulated by several transcription factors. *KLF4* and *FOXA1* were reported to form reinforcing regulatory loops with *SNAI2* in prostate cancer cell lines^15^. Another reciprocal transcriptional repression was reported between *SOX3* and *SNAI2*. This antagonistic relationship is involved in the regulation of subdivision of the early embryo into ectodermal and mesendodermal lineages^16^. A short splice variant of the Per-Arnt-Sim transcription factor Singleminded-2 (*SIM2*), has been shown to repress *SNAI2* in a dose-dependent manner in breast cancer cell lines^17^. *ELF5*, an ETS (E-twenty-six)-domain transcription factor which suppressed *SNAI2* during normal mammary gland development^18^.

Though being highly similar in terms of the structure, *SNAI1* and *SNAI2* have been shown to display context-dependent functional roles. Recent data have suggested that *SNAI1* and *SNAI2* are differentially expressed in normal mammary glands and in mammary tumours that distinctly induces EMT program. *SNAI1* occupies far more promoters than *SNAI2* does, suggesting a more exclusive role of *SNAI2*^19^. There is scattered information regarding how *SNAI1* and *SNAI2* regulate each other and how *SNAI1* and *SNAI2* is chosen under different contexts to execute exclusive functions.

In this study, we have reported the negative regulation of SNAI1 on *SNAI2* expression in ovarian cancer via the recruitment of the histone deacetylase (HDAC) corepressor to the proximal E-box binding sites.

## Results

### *SNAI1* expression shows negative correlation with *SNAI2*

**Figure 1:**
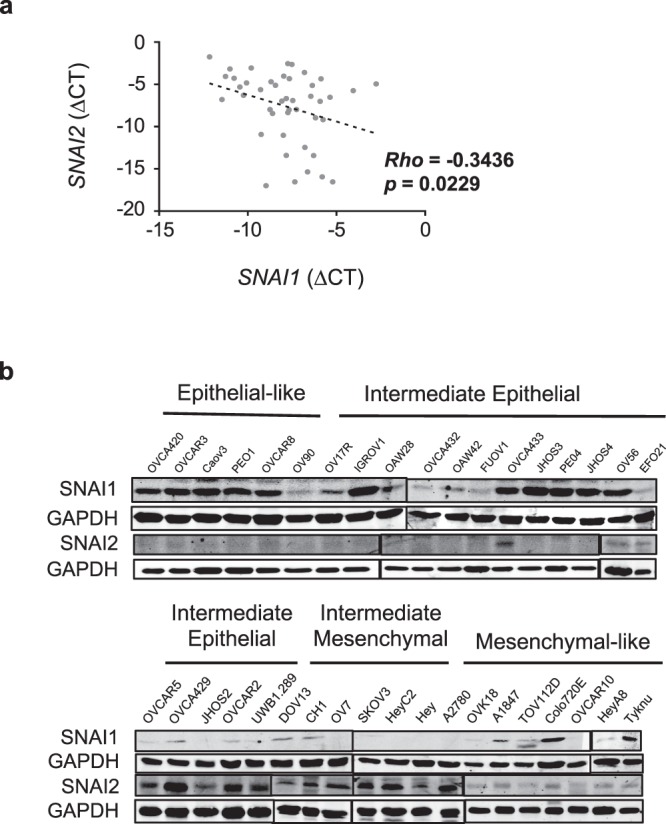
SNAI1 negatively correlates with SNAI2. (**a**) mRNA expression correlation between *SNAI1* and *SNAI2*, quantified through qRT-PCR in the SGOCL collection of ovarian cancer cell lines (*n* = 42). (**b**) Immunoblots showing expressions of SNAI1 & SNAI2 in 37 ovarian cancer cell lines representing four phenotypes of the EMT spectrum. GAPDH was used as a loading control.

By using an in-house panel of ovarian cancer cell lines, SGOCL, we firstly investigated the *SNAI1* and *SNAI2* mRNA expression and protein abundance. At the transcript level, there was a negative correlation (Rho = −0.3436; *p* = 0.0229) between *SNAI1* and *SNAI2* mRNA expressions across the SGOCL panel (Fig. 1a). The SGOCL panel was characterized into four phenotypes constituting the EMT spectrum: Epithelial (E), Intermediate Epithelial (IE), Intermediate Mesenchymal (IM) and Mesenchymal (M) and the delineation for each cell line was determined based on morphological examination and immunofluorescence staining of E-cadherin, Pan-cytokeratin and Vimentin^20^. The protein abundance of SNAI1 and SNAI2 in the SGOCL panel further showed a mutually exclusive pattern (Fig. 1b). With the exception of two mesenchymal cell lines, SNAI1 was predominantly expressed in cell lines with an epithelial-like phenotype (E and IE). In contrast, expression of SNAI2 was undetectable in epithelial-like (E), high expression in intermediate cell lines (IE and IM) and moderate expression in all cell lines with mesenchymal-like phenotype (M). To validate the presence of differential expression between SNAI1 and SNAI2 in other cancers, we subjected the lung adenocarcinoma cell line, A549 to TGFβ treatment. The results showed a gradual downregulation of epithelial marker, E-cadherin expression and simultaneous upregulation of mesenchymal genes *VIM*, *SNAI1* and *SNAI2* in all analysed time points (Supplementary Fig. 1). Notably, a trend of reciprocal expression of *SNAI1* and *SNAI2* was observed during the treatment.

### SNAI1 represses *SNAI2* expression

**Figure 2:**
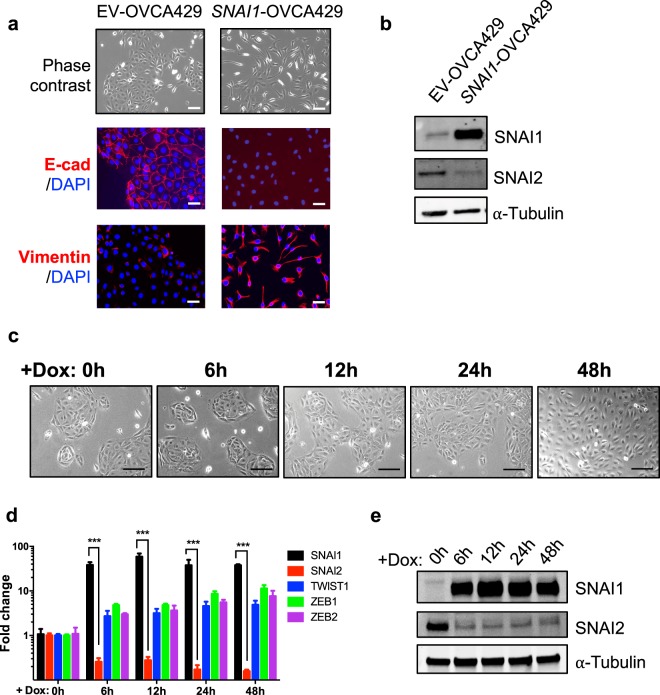
SNAI1 overexpression leads to downregulation of SNAI2. (**a**) Phase contrast images showing morphology of OVCA429 cells stably expressing control (EV) and full-length SNAI1 (SNAI1). Bottom panels showing immunofluorescence staining of E-cadherin and Vimentin in control and SNAI1 overexpressing cells. Nuclei were stained blue (DAPI), Scale = 50 μm. (**b**) Immunoblots showing expression of SNAI1 & SNAI2 in control and SNAI1 overexpressing cells. α-Tubulin was used as a loading control. (**c**) Phase contrast images of OVCA429 cells expressing control and SNAI1 cells at different time points after the addition of doxycycline (2 μg/ml), Scale = 50 μm. (**d**,**e**) Expression levels of indicated genes upon different time points after doxycycline treatment as analysed by qRT-PCR (**d**) and western blotting (**e**).

To confirm whether the correlation was an actual causal relationship, ovarian cancer cell line with epithelia-like phenotype, OVCA429 was engineered to overexpress SNAI1 with a stable GFP-tagged construct or a Tet-inducible system. Constitutively *SNAI1* overexpressing OVCA429 (*SNAI1*-OVCA429) cells showed a complete EMT phenotype (Fig. 2a) with a spindle-shaped morphology compared to the respective empty vector (EV) control cells. Immunofluorescence imaging of *SNAI1*-OVCA429 cells completely lost cell-cell adhesion protein E-cadherin expression and showed a profound increase in mesenchymal marker Vimentin expression, denoting the acquisition of dispersed fibroblastic-like morphology. In addition, downregulation of SNAI2 in *SNAI1*-OVCA429 cells was also evident at the protein level (Fig. 2b). Tet-induced *SNAI1* overexpressing OVCA429 cells displayed time-dependent morphological changes following doxycycline induction, towards more mesenchymal-like phenotypes (Fig. 2c). Quantitative PCR (qPCR) was performed to validate the overexpression of *SNAI1* and to investigate the cross-regulation of other EMT-TFs comparing to the 0 h controls. qPCR results confirmed that after Tet induction, OVCA429 cells consistently demonstrated *SNAI2* downregulation upon *SNAI1* overexpression (Fig. 2d,e). Interestingly, *TWIST1* and *ZEB1/2* were also upregulated upon *SNAI1* induction (Fig. 2d). *TWIST1* overexpression in OVCA429 cells did not consistently cause *SNAI2* repression (data not shown). Taken together, *SNAI1* directly represses *SNAI2* expression while other EMT transcription factors *TWIST1* and *ZEB1/2* had no major role in this system.

### SNAI1 represses *SNAI2* via recruiting HDAC to its proximal promoter region

**Figure 3:**
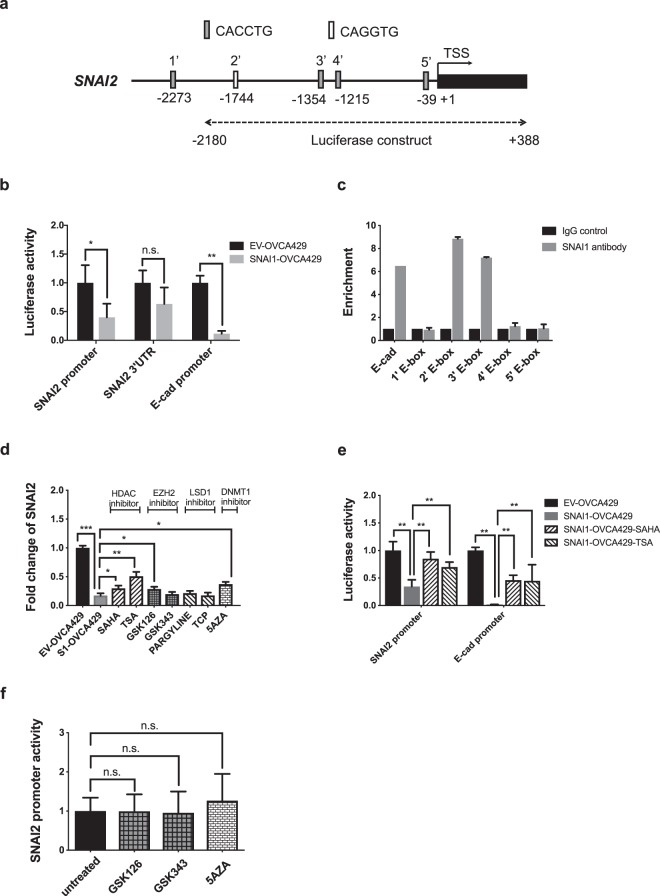
SNAI1 functionally represses SNAI2. (**a**) Schematic representation of the promoter region of human *SNAI2*, indicating putative SNAI1 binding E-box sequences. Numbers indicate positions in bps on chromosomal DNA relative to the transcription start site (+1). Nucleotide positions (in bps) cloned in to a luciferase construct for reporter assays were also indicated. (**b**) Luciferase activity of *SNAI2* promoter, *SNAI2* 3′UTR & *CDH1* (E-cad) promoter showing reduced activity in SNAI1-overexpressing OVCA429 cells compared to control cells. (**c**) ChIP-qPCR analysis of IgG (control) and SNAI1 in SNAI1-overexpressing OVCA429 cells showing enhanced enrichment of SNAI1 binding in 2′ and 3′ E-box sequences (indicated in A) of the SNAI2 promoter. SNAI1 binding E-box region of CDH1 (E-cad) promoter was used as a positive control. Signals were normalized to input DNA and plotted as enrichments relative to its respective IgG control (**d**) Fold change of *SNAI2* expression after inhibitors of HDAC, EZH2, LSD1 and DNMT1 corepressor complexes in SNAI1-overexpressing OVCA429 cells. (**e**) Luciferase activity of SNAI2 and CDH1 (E-cad) promoter regions (containing E-boxes) with or without HDAC inhibitor treatments in control and SNAI1-overexpressing OVCA429 cells. (**f**) Luciferase activity of SNAI2 promoter with or without EZH2 and DNMT1 inhibitor treatments in control and SNAI1-overexpressing OVCA429 cells.

To further elucidate how SNAI1 repressed *SNAI2*, promoter assays were developed. A 2-kb *SNAI2* promoter sequences containing four putative SNAI1 binding E-box sequences (Fig. 3a) were cloned into pGL3 vectors and transfected into both EV- and *SNAI1*-OVCA429. The full-length 3′UTR sequence of *SNAI2* (Supplementary Fig. 2) was cloned into a pGL3 vector as a negative control. An E-cadherin promoter containing the SNAI1 binding E-box sequences (Supplementary Fig. 3) was used as a positive control of the transcriptional repression function of *SNAI1*. From the promoter luciferase activity results (Fig. 3b), the *SNAI2* promoter luciferase activity was significantly lower in *SNAI1*-OVCA429 compared to EV-OVCA429. There was no significant change in the *SNAI2* 3′UTR luciferase activity. Consistent with its transcription repression role for E-cadherin, the E-cadherin promoter luciferase activity was significantly lower in *SNAI1*-OVCA429 compared to EV-OVCA429. There were five E-box sequences identified at the *SNAI2* 5′ promoter site (Fig. 3a). Chromatin immunoprecipitation (ChIP)-qPCR was utilized to identify which E-box sequence would be the putative SNAI1 binding site for *SNAI2* regulation. The SNAI1 binding site for E-cadherin was used as a positive control. Two of the five E-box sequences displayed enrichments of *SNAI1* binding compared to the IgG controls (Fig. 3c). These two E-boxes are located at −1744 and −1354 upstream from the transcription start site (TSS) of *SNAI2*. Our results thus suggested that SNAI1 repressed *SNAI2* transcription through direct binding at its proximal promoter.

SNAI1 was known to repress target gene expression through recruiting corepressors including HDACs, Polycomb repressive complex 2 (PRC2), Lysine-specific demethylase 1 (LSD1) and DNA methyltransferase 1 (DNMT1)^21,22^. Therefore, we utilized a series of inhibitors of these known corepressors to further elucidate the mechanism of *SNAI2* repression. *SNAI1*-OVCA429 was treated with HDAC inhibitors (TSA and SAHA), EZH2 inhibitors (GSK126 and GSK343), LSD1 inhibitors (Pargyline and TCP), and a DNMT1 inhibitor (5-AZA) to test the effects on *SNAI2* expression. Only the DNMT1 inhibitor (5-AZA), one of the EZH2 inhibitors (GSK126), and both HDAC inhibitors (TSA and SAHA), showed partial rescue of *SNAI2* expression (Fig. 3d). Among them, TSA showed the highest fold (up to 50%) of *SNAI2* rescue expression. The LSD1 inhibitors had no effect to restore *SNAI2* expression in *SNAI1*-OVCA429. We utilized the *SNAI2* promoter activity to further confirm our results. Only HDAC inhibitor-treated *SNAI1*-OVCA429 showed a significant increase of *SNAI2* and E-cadherin promoter activities (Fig. 3e). EZH2 and DNMT1 inhibitors showed no effect on the *SNAI2* promoter activity (Fig. 3f). These results indicated that the recruitment of HDAC by SNAI1 might be directly responsible for the *SNAI2* repression. The recruitment of other corepressors such as DNMT1 and EZH2 to the *SNAI2* site might be secondary.

### Change of histone marks and chromatin structure at the *SNAI2* promoter region in SNAI1 overexpressing cells

**Figure 4:**
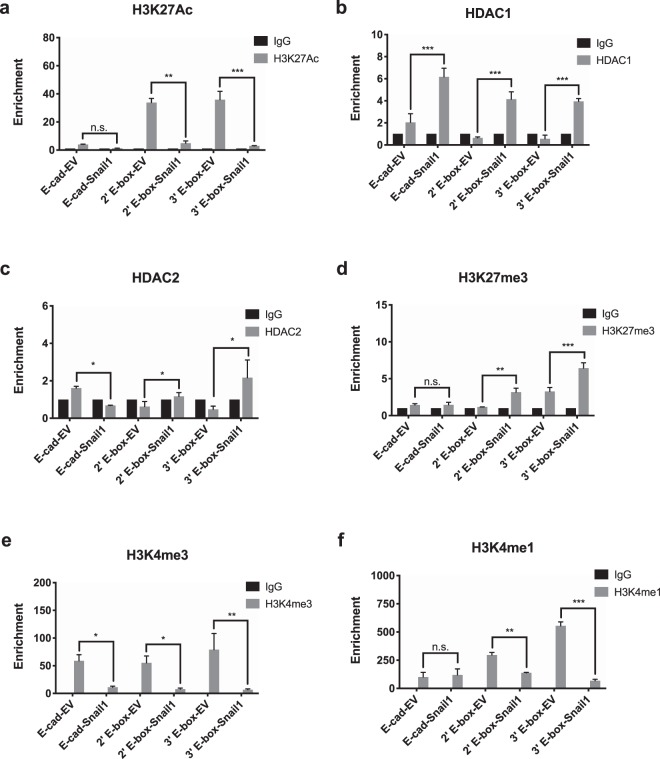
Alterations of histone marks and chromatin landscape at the *SNAI2* promoter. ChIP-qPCR analysis of IgG (control) and H3K27Ac (**a**), HDAC1 (**b**), HDAC2 (**c**), H3K27me3 (**d**), H3K4me3 (**e**) and H3K4me1 (**f**) in SNAI1 binding 2′ and 3′E-box sequences of the SNAI2 promoter. SNAI1 binding E-box region of CDH1 (E-cad) promoter was used as a positive control. Signals were normalized to input DNA and plotted as enrichments relative to its respective IgG control. Statistical significance were calculated by comparing the enrichments between EV (control) and SNAI-overexpressing OVCA429 cells.

Knowing that HDAC activity is required for *SNAI2* repression by SNAI1, the histone marks and chromatin structure at the *SNAI2* locus following SNAI1 overexpression were further explored. Since SNAI1 was reported to modify chromatin histone marks, enrichments of histone acetylation mark H3K27Ac and methylation marks H3K27me3, H3K4me1 and H3K4me3 at the 2^nd^ and 3^rd^ E-box regions of SNAI2 promoter were investigated in the EV- and SNAI1-OVCA429 cells. Abundant enrichment of H3K27Ac (open promoter) was found in the EV-OVCA429 cells (Fig. 4a) compared to the SNAI1-OVCA429 cells, denoted that chromatin at these E-box positions could be deacetylated for transcriptional repression after SNAI1 overexpression. Among the HDACs, HDAC1 and HDAC2 were known to interact with SNAI1 to repress transcription of E-cadherin^8^. Accordingly, we observed significant enrichments of HDAC1 and HDAC2 binding in both E-box positions of *SNAI2* promoter, exclusively in *SNAI1*-OVCA429 cells (Fig. 4b,c). In addition, there was a significant enrichment of repressive histone mark, H3K27me3 on both E-box regions of *SNAI2* promoter only in *SNAI1*-OVCA429 cells (Fig. 4d). In the same cells, there were significant reductions in active H3K4 mono- and tri-methylation histone marks in both E-box segments, indicated that these regions were inaccessible after *SNAI1* overexpression (Fig. 4e,f). Collectively, these data suggested that induction of SNAI1 expression in cells dramatically altered chromatin histone marks along the *SNAI2* promoter region, which ultimately contributed to efficient repression of *SNAI2* expression.

## Discussion

In this study, we showed that SNAI1 suppressed *SNAI2* expression through direct promoter binding and this repressive activity required recruitment of transcriptional corepressor HDAC complex. In addition, we identified that SNAI1 association on *SNAI2* promoter generated a dramatic change in chromatin organization that further influenced the expression on SNAI2 in cell-type specific states.

Our protein abundance profiling of SNAI1 and SNAI2 in the SGOCL panel showed that SNAI1 was predominantly expressed in cell lines with epithelial phenotype. In contrast, none of the cell lines with epithelial phenotype showed endogenous expression of SNAI2. At first, this mutually exclusive expression might have denoted that SNAI1 efficiently repressed SNAI2 expression. However, the expression of E-cadherin (regarded as primary target of SNAI1) in the same panel was unaffected^23^. This discrepancy could be partly explained by previous studies that showed repression of E-cadherin by SNAI1 was dependent on the activity of Polycomb repressive complex 2 (PRC2), histone methyltransferase Suv39H1 and histone demethylase LSD1^10,11,24^. Whether our panel of epithelial cancer lines lacked LSD1, PRC2 and Suv39H1 activity or expression remains to be investigated. Moreover, the observed negative correlation of *SNAI1* and *SNAI2* in the SGOCL panel was evident at the transcript level, that led us to further investigate the mechanism of *SNAI2* repression in *SNAI1* expressing cells.

Recent findings clearly highlighted that EMT in cancer was a dynamic process with acquisition of molecular and phenotypical changes along multiple distinct intermediate phases^20,25,26^. Supporting to these findings, we observed that overexpression of *SNAI1* in a epithelia-like cell line (OVCA420) generated only a partial EMT like changes (intermediate epithelial phenotype) and retained cell-cell adhesion (data not shown). Intriguingly, *SNAI1* overexpression in intermediate epithelial cell line (OVCA429) driven the cells to a near-complete EMT like phenotype that completely lost cell-cell adhesion, leading to drastic rearrangement of cellular morphology. This clearly demonstrated that even a strong EMT inducing transcription factor such as SNAI1 operates differentially in each cell type belonging to different EMT status. Inducible expression of mouse *Snail* (mSnail) in MDCK cells modulated the levels of specific claudins but the epithelial tight junction organization remained unaltered^27^. In contrast, E-cadherin localization at the cell-cell contact became undetectable after the expression of mSnail in mouse epithelial cell line, Eph4^28^. Therefore, it was evident that EMT induced through SNAI1 in cells were context-dependent and possibly have driven the cells to the next intermediary state close to a mesenchymal phenotype.

Moreover, completion of EMT could be attained through cooperative functioning of different transcription factors such as SNAI1/2, TWIST and ZEB1/2. Among which, SNAI1 was reported to express at the onset of EMT and subsequently other EMT factors were induced at later time points to strengthen a mesenchymal state^3,29,30^. Accordingly, in our Tet-inducible system, induction of *SNAI1* at regular intervals displayed a consistent upregulation of *TWIST1*, *ZEB1/2* and simultaneous downregulation of *SNAI2* expression. The observed regulation potentially driven the cells towards a mesenchymal (near complete EMT) morphology. Similar observation was reported using human mammary epithelial cells, where induction of *SNAI1* led to repression of *SNAI2* and simultaneous induction of *ZEB1/2* transcripts at an early stage, highlighting that execution of this functional circuit preceded other molecular and phenotypical changes that led to EMT^31^.

Earlier studies reported that SNAI1 and SNAI2 displayed a reciprocal expression in oral, breast cancer cells or during reprogramming of induced pluripotent stem cells^32–34^. In addition, another study showed that during mouse chondrogenic differentiation, endogenous SNAI1 and SNAI2 bind to their own and each other’s promoter^35^. However, none of these studies looked into the SNAI1 occupancy in *SNAI2* promoter and delineated the molecular mechanism that controlled this repressive regulation. Through luciferase reporter activity and chromatin immunoprecipitation assays we clearly showed that SNAI1 predominantly occupied two of the E-box sequences in *SNAI2* promoter region for transcriptional repression. Epigenetic repression of SNAI1 on its target genes have been carried out through recruitment of HDAC1/2 and corepressor Sin3A on its SNAG domain^8,36^. Supporting to these studies, our *SNAI2* promoter assays, HDACi treatments and chromatin immunoprecipitation results indicated the involvement of HDACs in SNAI1 mediated *SNAI2* suppression. However, further evidence has to be provided to elucidate whether HDACs were recruited to the *SNA12* promoter directly or not. In addition, the alterations of related acetylation markers of Histone H3 at the SNAI1 binding sites would be required.

In addition to HDACs, SNAI1 also recruited other chromatin modifying enzymes including DNMT1, PRC2, LSD1, G9a and Suv39H1 at the E-cadherin promoter for transcriptional silencing^21,37^. Our results showed that the dissociation of active methylation marks and enhancement of repressive methylation marks along the SNAI1 binding *SNAI2* promoter region. However, only DNMT1 inhibitor treatment could partially rescue the re-expression of *SNAI2*, indicated that epigenetic regulation of SNAI1 at the *SNAI2* promoter region is primarily executed through the histone deacetylation complexes.

The expression of HDAC family members along the different histological subtypes of ovarian cancer showed a wide heterogeneity^38^. Therefore, utilization of HDAC inhibitors with or without conventional chemotherapy to treat ovarian cancer needs further investigations. Recently, ovarian cancer cells carrying ARID1A mutation showed higher sensitivity to SAHA and ACY1215 treatment, through inhibition of HDAC2 and HDAC6 activity respectively^39,40^. On a another note, SNAI1 expression was high in majority of ovarian cancer, except mucinous and clear cell carcinoma^41^. Therefore, a strategic method to segregate patients harbouring SNAI1 and HDAC dependency during tumour progression is essential to efficiently use existing HDAC inhibitors as cancer therapeutics.

## Methods

### Generation of stable and inducible cell lines

For stable overexpression of SNAI1, plasmid encoding full length wide type SNAI1 cloned from pCMV-Entry-SNAI1 (Origene) into pLenti-GIII-CMV-GFP-2A-Puro vector (ABM). Empty vector with no inserts was used as negative control. Plasmids were mixed with Mission Lentiviral Packaging Mix (Sigma-Aldrich) before added to a mixture of transfection reagent Fugene 6 (Roche). After 15 minutes incubation at room temperature, plasmid mix was added to 293T cells and viral supernatants were harvested at 48 and 72 hours post-transfection. Cells infected with lentivirus were selected using puromycin at a proper concentration decided by their respective puromycin kill curve.

To generate Tet-inducible SNAI1 expressing cells, SNAI1 was cloned from pCMV6-Entry-SNAI1 vector using into pLVX-TRE3G vector (Clontech). pLVX-TRE3G-SNAI1 was cotransfected with pLVX-EF1Alpha-Tet3G (Clontech) to 293T cells to generate lentiviral supernatants. Cells infected with lentivirus were dually selected using puromycin and G418. Same primer pairs containing *BamHI* and *EcoRI* were used for generating both vectors and were listed in Supplementary Table S1.

### Quantitative real-time PCR (qRT-PCR) and ChIP-qPCR

Total RNA from Tet-inducible SNAI1 overexpressing OVCA429 cells and corepressor inhibitor treated cells were extracted using RNeasy mini kit (SAbiosciences, Qiagen) according to manufacturer’s protocol. mRNA was reverse-transcribed to cDNA using RT2 first strand kit (SAbiosciences, Qiagen) and cDNA was mixed with SYBR green master mix (SAbiosciences, Qiagen) for qPCR analysis. Five housekeeping genes ACTB, B2M, GAPDH, HPRT1 and RPL13A were used for normalization and used as previously described^23^. mRNA expression level was presented as average fold change (2^−∆∆Ct^) with respect to control, from minimum two biological replicates. All qPCR experiments were done using ABI 7900HT (Life Technologies).

Chromatin immunoprecipitation (ChIP) were performed as described previously with following modifications^23^. Sheared chromatin was incubated with IgG (sc-2028, Santa Cruz), SNAI1 (sc-10432, Santa Cruz), H3K27Ac, H3K27me1, H3K3me1, H3K4me3, HDAC1 and HDAC2 antibodies. Crosslinked DNA was eluted using QIAquick PCR purification kit (Qiagen) following manufacturer’s protocol. Following DNA purification, primers spanning E-box regions of SNAI2 and E-cad promoters were used (listed in Supplementary Table S2) and were amplified by qRT-PCR. Fold enrichments were calculated relative to respective IgG controls, from at least two biological replicates.

### Western blot analysis

An ovarian cell line library, referred as SGOCL, comprising of 43 different ovarian cancer cell lines were generated from different sources as described previously^20^. Whole cell protein cell lysates for 37 cell lines of the SGOCL were extracted as described previously^23^. Other cell lysates were harvested using RIPA buffer and resolved by standard reducing SDS-PAGE followed by blotting on PVDF membranes. Immunoblots were incubated with appropriate antibodies, anti-SNAI1 (C15D3, CST); anti-SNAI2 (C19G7, CST); anti-α-Tubulin (DM1A, Abcam) diluted in 2% BSA in PBS. Blots were scanned using the Odyssey Infrared Imaging System (Li-COR). Images were transferred to gray scale.

### Immunofluorescence staining

Cells were fixed with 4% paraformaldehyde and blocked with 3% BSA/PBS. Fixed cells were incubated with primary antibodies: anti-E-cadherin (61018, BD Biosciences) and secondary antibody conjugated with Alexa Fluor 594 (Invitrogen). F-actin was stained by Rhodamine phalloidin (R415, Life Technologies). Stained cover slips were mounted onto glass slides using Vectashield mounting medium containing DAPI (#H-1200, Vector Laboratories).

### Molecular cloning

The 2569 bp length of *SNAI2* promoter segment, 658 bp length of *CDH1* promoter regions and were cloned in to pGL3 Luciferase reporter vector (Promega) using primers containing *KpnI* and *XhoI* restriction sites. Primers used for cloning were listed in Supplementary Table S1.

### Promoter assay

Plasmid transfections were carried out using transfection reagent Fugene 6 (Roche) following manufacturer protocol. EV-OVCA429 and SNAI1-OVCA429 cells were transfected with 100 ng of pGL3-SNAI2-promoter/pGL3-SNAI2-3′UTR/pGL3-Ecadherin-promoter together with 1.5 ng of pCMV-renilla. 24 hours post transfection, Firefly and Renilla luciferase activities were measured using Dual-Glo Luciferase assay system (Promega).

### Statistical analyzes

To analyze ChIP data, multi-group comparisons were performed using two-way ANOVA and Tukey’s *post hoc* test if necessary. For other analyzes, two-tailed Student’s *t*-test was used to access statistical significance. For correlation analysis, Pearson correlation was performed. *p ≤ 0.05; **p ≤ 0.01; ***p ≤ 0.001; n.s. not-significant.

## Supplementary information

Supplementary info 1

## Data Availability

Datasets used in the current study are available upon request from the corresponding author.
