# Supplementary material for: SNAI1 recruits HDAC1 to suppress SNAI2 transcription during epithelial to mesenchymal transition
Source: Sci Rep. 2019 Jun 5;9:8295. doi: 10.1038/s41598-019-44826-8 (PMC6549180; doi:10.1038/s41598-019-44826-8)
Supplement: Supplementary file 1 — Supplementary info 1 [file 41598_2019_44826_MOESM1_ESM.docx]

**SNAI1 recruits HDAC1 to suppress *SNAI2* transcription during epithelial to mesenchymal transition**

Vignesh Sundararajan^1#^, Ming Tan^1#^, Tuan Zea Tan^1^, Jieru Ye^1^, Jean Paul Thiery^2,3,4,5^, Ruby Yun-Ju Huang^1,6,7, a *^

#These authors contributed equally to this work

*Corresponding author: Ruby Yun-Ju Huang, email: rubyhuang@ntu.edu.tw, phone: +886 23123456 ext. 88399

^a^Current address: School of Medicine, College of Medicine, National Taiwan University, No.1 Ren Ai Road Section 1, Taipei, 10051, Taiwan, Republic of China

^1^Cancer Science Institute of Singapore, Singapore; National University of Singapore, Center for Translational Medicine, 14 Medical Drive, MD6 #12-01, Singapore 117599

^2^Department of Biochemistry Yong Loo Lin School of Medicine, National University of Singapore, 8 Medical Drive, MD7, #02-03, Singapore 117597

^3^Guangzhou Institute of Biomedicine and Health, Chinese Academy of Science, People’s Republic of China

^4^CNRS Emeritus CNRS UMR 7057 Matter and Complex Systems, University Paris Denis Diderot, Paris, France

^5^INSERM UMR 1186, Integrative Tumor Immunology and Genetic Oncology, Gustave Roussy, EPHE, PSL, Fac. de Médecine - Univ. Paris-Sud, Université Paris-Saclay, 94805, Villejuif, France.

^6^Department of Obstetrics and Gynaecology, National University Hospital of Singapore, Singapore, 1E Kent Ridge Road Singapore 119228

^7^Department of Anatomy, Yong Loo Lin School of Medicine, National University of Singapore, 4 Medical Drive, MD10 #04-01, Singapore 117597

**Supplementary Figure 1. Effects of TGFβ treatment in A549 cells.**

The mRNA expression of *CDH1, VIM, SNAI1* and *SNAI2* analyzed by qRT-PCR in untreated and TGFβ treated cells at indicated time points. The fold change (2^-∆∆Ct^) was calculated with respect to untreated cells.

**Supplementary Figure 2. Full length 3’UTR of SNAI2.**

Primers used for cloning highlighted in gray.

**>SNAI2_3’UTR**

TTGCCCTCACTGCAACAGAGCATTTGCAGACAGGTCAAATCTGAGGGCTCATCTGCAGACCCATTCTGATGTAAAGAAATACCAGTGCAAAAACTGCTCCAAAACCTTCTCCAGAATGTCTCTCCTGCACAAACATGAGGAATCTGGCTGCTGTGTAGCACACTGAGTGACGCAATCAATGTTTACTCGAACAGAATGCATTTCTTCACTCCGAAGCCAAATGACAAATAAAGTCCAAAGGCATTTTCTCCTGTGCTGACCAACCAAATAATATGTATAGACACACACACATATGCACACACACACACACACACCCACAGAGAGAGAGCTGCAAGAGCATGGAATTCATGTGTTTAAAGATAATCCTTTCCATGTGAAGTTTAAAATTACTATATATTTGCTGATGGCTAGATTGAGAGAATAAAAGACAGTAACCTTTCTCTTCAAAGATAAAATGAAAAGCACATTGCATCTTTTCTTCCTAAAAAAATGCAAAGATTTACATTGCTGCCAAATCATTTCAACTGAAAAGAACAGTATTGCTTTGTAATAGAGTCTGTAATAGGATTTCCCATAGGAAGAGATCTGCCAGACGCGAACTCAGGTGCCTTAAAAAGTATTCCAAGTTTACTCCATTACATGTCGGTTGTCTGGTTGCCATTGTTGAACTAAAGCCTTTTTTTGATTACCTGTAGTGCTTTAAAGTATATTTTTAAAAGGGAGGAAAAAAATAACAAGAACAAAACACAGGAGAATGTATTAAAAGTATTTTTGTTTTGTTTTGTTTTTGCCAATTAACAGTATGTGCCTTGGGGGAGGAGGGAAAGATTAGCTTTGAACATTCCTGGCGCATGCTCCATTGTCTTACTATTTTAAAACATTTTAATAATTTTTGAAAATTAATTAAAGATGGGAATAAGTGCAAAAGAGGATTCTTACAAATTCATTAATGTACTTAAACTATTTCAAATGCATACCACAAATGCAATAATACAATACCCCTTCCAAGTGCCTTTTTAAATTGTATAGTTGATGAGTCAATGTAAATTTGTGTTTATTTTTATATGATTGAATGAGTTCTGTATGAAACTGAGATGTTGTCTATAGCTATGTCTATAAACAACCTGAAGACTTGTGAAATCAATGTTTCTTTTTTAAAAAACAATTTTCAAGTTTTTTTTACAATAAACAGTTTTGATTTAAAATCTCGTTTGTATACTATTTTCAGAGACTTTACTTGCTT

**Supplementary Figure 3. SNAI1 binding regions on CDH1 promoter.**

Primers used for cloning highlighted in gray. E-boxes were indicated in bold red font.

**>CDH1_promoter**

TCTACAAAAAGGCAAAAGAAAAAAAAATTAGCCTGGCGTGGTGGTGTG**CACCTG**TACTCCCAGCTACTAGAGAGGCTGGGGCCAGAGGACCGCTTGAGCCCAGGAGTTCGAGGCTGCAGTGAGCTGTGATCGCACCACTGCACTCCAGCTTGGGTGAAAGAGTGAGACCCCATCTCCAAAACGAACAAACAAAAAATCCCAAAAAACAAAAGAACTCAGCCAAGTGTAAAAGCCCTTTCTGATCCCAGGTCTTAGTGAGCCACCGGCGGGGCTGGGATTCGAACCCAGTGGAATCAGAACCGTGCAGGTCCCATAACCCACCTAGACCCTAGCAACTCCAGGCTAGAGGGTCACCGCGTCTATGCGAGGCCGGGTGGGCGGGCCGTCAGCTCCGCCCTGGGGAGGGGTCCGCGCTGCTGATTGGCTGTGGCCGG**CAGGTG**AACCCTCAGCCAATCAGCGGTACGGGGGGCGGTGCCTCCGGGGCT**CACCTG**GCTGCAGCCACGCACCCCCTCTCAGTGGCGTCGGAACTGCAAAG**CACCTG**TGAGCTTGCGGAAGTCAGTTCAGACTCCAGCCCGCTCCAGCCCGGCCCGACCCGACCGCACCCGGCGCCTGCCCTCGCTCGGCGTCCCCGGCCAGCCATGGGCCCTTGGAGCCGCAG

**Supplementary Figure 4. Full-length blots.**


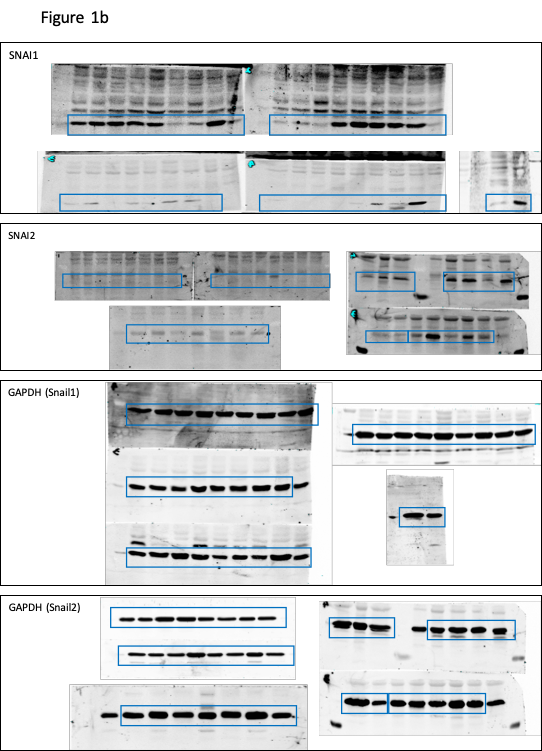


**Supplementary Figure 4 (continued)**

**Supplementary Table S1. Primers used for cloning.**

*BamHI* site in forward primer and *EcoRI* site in reverse primer were highlighted in gray.

SNAI1 full-length (forward) TCTGGGATCCCGATCGCCATGCCGCGCTCTTT

(reverse) GCCGGAATTCTTAGCGGGGACATCCTGAGCAG

*KpnI* site in forward primer and *XhoI* site in reverse primer were highlighted in gray.

SNAI2 promoter (forward) ATCGGGTACCGCACGCGGATCTGTGTAATG

(reverse) ATCGCTCGAGGCAAACGAAGCTGCGAGATTT

SNAI2 3’UTR (forward) AGCCGGTACCTTGCCCTCACTGCAAC

(reverse) ACTACTCGAGAAGCAAGTAAAGTCTCT

CDH1 promoter (forward) GACTGGTACCTCTACAAAAAGGCAAAAGAAAA

(reverse) CGCCCTCGAGCTGCGGCTCCAAGGGCCCATGG

**Supplementary Table S2. Primers used for ChIP-qPCR.**

SNAI2 promoter:

1’E-box (forward) CGGCTTGCGTTTTTACCACA

(reverse) AGCGAGTAACACGTATGCCC

2’E-box (forward) GCCATGGCGATATGTGTTTTCTC

(reverse) GCCAGAGGCAGTTTTCTAAGC

3’E-box (forward) TTAGGAAATCTGTGAGTGCCCC

(reverse) TCACATGAAGATCACCCTACTCTT

4’E-box (forward) AGCACCTGTTAGAAACAAGAGT

(reverse) ACCCAGTCTAGTAACTGCAAAAAC

5’E-box (forward) CGTAAAGGAGCCGGGTGAC

(reverse) TGTGTGTCCAGTTCGCTGTAG

E-cadherin promoter:

E-box (forward) ACTCCAGGCTAGAGGGTCACC

(reverse) CCGCAAGCTCACAGGTGCTTTGCAGTTCC
